# Cyclophilins and Their Functions in Abiotic Stress and Plant–Microbe Interactions

**DOI:** 10.3390/biom11091390

**Published:** 2021-09-21

**Authors:** Przemysław Olejnik, Cezary Jerzy Mądrzak, Katarzyna Nuc

**Affiliations:** Department of Biochemistry and Biotechnology, Poznań University of Life Sciences, Dojazd 11, 60-632 Poznań, Poland; przemyslaw.olejnik@up.poznan.pl (P.O.); cezary.madrzak@up.poznan.pl (C.J.M.)

**Keywords:** cyclophilins, peptide bond isomerization, stress, pathogenesis, plant–microbe interaction

## Abstract

Plants have developed a variety of mechanisms and regulatory pathways to change their gene expression profiles in response to abiotic stress conditions and plant–microbe interactions. The plant–microbe interaction can be pathogenic or beneficial. Stress conditions, both abiotic and pathogenic, negatively affect the growth, development, yield and quality of plants, which is very important for crops. In contrast, the plant–microbe interaction could be growth-promoting. One of the proteins involved in plant response to stress conditions and plant–microbe interactions is cyclophilin. Cyclophilins (CyPs), together with FK506-binding proteins (FKBPs) and parvulins, belong to a big family of proteins with peptidyl-prolyl *cis-trans* isomerase activity (Enzyme Commission (EC) number 5.2.1.8). Genes coding for proteins with the CyP domain are widely expressed in all organisms examined, including bacteria, fungi, animals, and plants. Their different forms can be found in the cytoplasm, endoplasmic reticulum, nucleus, chloroplast, mitochondrion and in the phloem space. They are involved in numerous processes, such as protein folding, cellular signaling, mRNA processing, protein degradation and apoptosis. In the past few years, many new functions, and molecular mechanisms for cyclophilins have been discovered. In this review, we aim to summarize recent advances in cyclophilin research to improve our understanding of their biological functions in plant defense and symbiotic plant–microbe interactions.

## 1. Introduction

Cyclophilins constitute a group of proteins with peptidyl-prolyl *cis-trans* isomerase activity (PPIase) involved in the folding of target proteins; they catalyze the reaction in both directions [1,2,3,4]. Peptide bonds are most probably synthesized by ribosomes in the *trans* configuration, and *cis* peptidyl-prolyl bonds are present at a small percentage in proteins [5]. Isomerization around Xaa-Pro bonds is one of the most rate-limiting steps in protein folding. These isomerases also belong to the family of immunophilins, which consists of two groups—cyclophilins (CyPs) and FK506-binding proteins (FKBPs)—initially discovered as receptors for immunosuppressive drugs in mammals [6,7]. Another group of proteins with PPIase activity is parvulins, but they do not belong to the immunophilin family since they are not sensitive to any specific immunosuppressive drug [8]. Parvulins regulate the structure and function of eukaryotic RNA polymerase II catalyzing the *cis-trans* conversion of Ser5–Pro6 bonds within the carboxy–terminal domain (CTD) repeats, of the largest subunit of RNA polymerase II [9,10,11]. These three groups of proteins share little sequence homology but have in common the peptidyl–prolyl *cis-trans* isomerase activity involved in protein folding processes. In addition to their basic function in protein folding, cyclophilins participate in many other cellular processes, such as signaling, nucleic acid interactions, mRNA processing and spliceosome assembly, protein degradation, and apoptosis, as well as in response to different stress stimuli [12,13,14,15,16,17,18]. In plants, cyclophilins control transcription, hormone signaling, plant development and interactions with pathogens [3,19,20]. For example, *Arabidopsis thaliana* AtCyP71 is involved in processes of chromatin remodeling and histone modification; it also interacts with histone H3 and regulates gene expression patterns that determine plant organogenesis [21,22]. *A. thaliana* AtCyP40 is involved in the interaction with AGO1, or an AGO1-interacting protein promoting miRNA-mediated gene repression [23]. It has been shown that some CyP transcripts accumulate in response to wounding, heat shock, and low temperature treatment as well as in symbiotic plant–microbe interactions [24,25,26,27,28].

## 2. Cyclophilin Domain Architecture 

Cyclophilins can be divided into two groups of proteins: single domain and modular cyclophilins. Single domain members contain a basic catalytic domain (cyclophilin domain) of about 120 amino acids (17 kDa) with peptidyl–prolyl *cis-trans* isomerase activity. The cyclophilin domain, responsible for the CsA binding also called CBD domain (CsA binding domain) is remarkably conserved in all genera. Some of the single domain cyclophilins have also signal sequences directing to a specific subcellular compartment. Modular cyclophilins contain, besides the CyP, also other domains. For example, in *A. thaliana*, AtCyP40 has CyP domain and the tetratricopeptide repeat domain (TPR); AtCyP59 contains a CyP domain followed by an RNA recognition motif (RRM) and a C-terminal domain enriched in charged amino acids [29]. The biggest known protein with the CyP domain is nucleoporin Nup358, which contains a leucine-rich region, four potential Ran binding sites flanked by nucleoporin-characteristic FXFG repeats, eight zinc finger motifs and a C-terminal CyP domain [30]. Many plant modular cyclophilins have domains characteristic of nuclear proteins involved in pre-mRNA maturation, such as AtCyP57, AtCyP59, AtCyP63 and AtCyP95 [8]. These characteristic domains are rich in arginine/serine (RS) and arginine/lysine (RK) dipeptides, typical for splicing factors called SR proteins [31,32]. In 1991, Walsh and coworkers determined the structure of the recombinant cyclophilin A from human T cells. This structure consists of three α-helices, eight β strands, and six β turns. Its globular shape is formed by an eight-stranded antiparallel beta-barrel structure, with two alpha-helices sitting on the top and the bottom, closing the barrel [33]. The center of the barrel is filled with hydrophobic residues constituting a so-called hydrophobic pocket, which is the binding site for proline-containing substrates and CsA [34]. The peptidyl-prolyl-cis/trans-isomerase domain of modular cyclophilins has the same folding as that of the single domain CyPs. It was also found that some plant CyPs (e.g., *A thaliana* TLP40) are the potential target protein of chloroplast disulfide oxidoreductase, thioredoxin (Trx) [35,36]. The peptidyl-prolyl *cis-trans* isomerase activity of cyclophilin is fully inactivated in the oxidized form, and its reduction by Trx recovers it. Plant genomes have a large number of genes encoding different isoforms of cyclophilins, e.g., 31 in *A. thaliana* (48 putative proteins), 29 in *Oryza sativa* (46 putative proteins) and in *Glycine max* 62 genes and proteins, while in the human genome there are 17 (19 different proteins) and in *Saccharomyces cerevisiae* 8 genes and proteins [37,38]. These results suggest that alternative splicing during cyclophilin’s gene expression may result in different protein products localized, for example, in different cellular compartments playing different functions. *A. thaliana* AtCyP20-3 has three splicing forms (AtCyP20-3a – c) which differ in their size and localization (chloroplasts or endoplasmic reticulum). It was shown that this cyclophilin is important for light and oxidative stresses [36]. The expression of genes encoding some plant cyclophilins is induced by environmental stress conditions, pathogenesis and plant-microbe interaction. 

## 3. Cyclophilin Function in Abiotic Stress

Abiotic stress factors, such as drought, salinity, heavy metals, strong light and extrema temperatures, are one of the main causes of plant growth and development downturn. Moreover, abiotic stress is a major cause of crop yield losses and can reduce the yield of crop plants by 50–70% [39,40]. Plants as sessile organisms develop regulatory pathways to change their gene expression profiles in response to temporal unfavorable environmental conditions [41,42,43,44,45]. Research conducted for several decades shows that cyclophilins are one of the protein families whose expression is changed in response to abiotic stress [46,47,48,49]. Information about abiotic stress-responsive cyclophilins are collected in Table 1.

### 3.1. Water Stress

Drought is an abiotic stress factor that has a huge impact at various levels of plants organization. Prolonged drought causes reduction in plant cells’ turgor, leading to an increase in the dissolved substances concentration in cytosol. Furthermore, drought causes a stomata closure, leading to the limitation of gas exchange, transpiration, and photosynthesis. However, plants can adapt to drought conditions by diverse biochemical and physiological changes, including modification of gene expression profile. Some research conducted on gene expression changes under drought stress shows that cyclophilins may take part in the plant response to water shortage.

One of the earliest plant reactions for water loss is stomata closure [69]. Stomata are a major “gate” that enables gas exchange and transpiration. They are surrounded by two specified guard cells, which, by turgor change, may close or open the stomata in response to variable environmental conditions [70]. At the cellular level, abscisic acid (ABA) is the main factor responsible for stomata closure [71]. Under water deficiency conditions, ABA production in leaf vascular increases and hormone is transported to guard cells, where it activates S-type anion channels, leading to ion efflux and a decrease in the guard cells turgor [72,73,74]. Furthermore, ABA accumulation in guard cells leads to activation of the NADPH oxidase and generation of the reactive oxygen species (ROS), which are secondary messengers in stomata closure triggered by this phytohormone. A higher level of ROS increases NO and calcium levels in cells, which leads to the activation of plasma membrane ion channels, ion efflux, and in consequence, stomata closure [75,76,77]. However, high ROS concentration may cause oxidative stress, which leads to cell damage. To control ROS homeostasis, cells possess certain antioxidants systems, which include glutathione and ascorbate peroxidases, superoxide dismutase, and catalase [53,78,79,80]. Liu and coworkers have shown that the expression level of the *ROC3* gene coding AtCyP19-1 cyclophilin elevates in response to drought stress and ABA treatment. Furthermore, AtCyP19-1 takes part in the stomata closure, inhibiting catalase activity, thereby maintaining an adequate ROS level in guard cells to ensure stomata closure [81]. Moreover, in an experiment with insertional *roc3* mutant, the authors observed inhibition of the stomata closure and lower expression of ABA-induced genes involved in plant dehydration stress, such as RD29A, RD29B, RAB18, ABI5, ABF2, ABF3, ERD10 and COR47, in comparison with WT plants [81,82,83]. The ski-interacting protein (SKIP) is a spliceosomal component and transcriptional regulator that modulates the abiotic stress signaling pathway [57,58,84,85,86]. It was shown that rice cyclophilin OsCyP18-2 interacts with SKIP (bounding with amino acids 56-95 of OsSKIP). Furthermore, drought induces similar expression of *OsSKIP* and *OsCyP18-2*, which supports the thesis that these proteins act together in response to stress. OsCyP18-2 interaction with SKIP enables this cyclophilin translocation into the nucleus. Since SKIP is a component of the spliceosome, it was suggested that complex OsSKIP-OsCyP18-2 takes part in mRNA transcription and pre-mRNA processing [60]. A. thaliana SKIP can interact with AtCyP18-2 and OsCyP18-2. The overexpression of *OsCyP18-2* in transgenic rice and A. thaliana increases drought tolerance and changes the expression and splicing patterns of stress-related genes in Arabidopsis under drought conditions. These findings suggest the important role of OsSKIP and OsCyP18-2 interactions in the transcriptional and post-transcriptional regulation of stress-related genes [60]. Kim and coworkers observed over 215-fold higher expression of the *AtCyP21-2* gene, compared to WT plants in tree calreticulin (*CRT*) genes’ knock-out mutants. CRT is an ER-localized Ca^2+^-binding protein whose expression is upregulated in ER stress. It was shown that knock-out mutants of three *CTR* genes or *AtCyP21-2* gene appear to be more sensitive to drought stress. The authors concluded that AtCyP21-2 may also participate in the protein-folding process during ER stress in association with calreticulins [68] In *O. sativa* plants, the OsCyP2-P cyclophilin encoding gene is upregulated in response to drought stress. Ectopic expression of *OsCyP2-P* in tobacco plants has a positive effect on seed germination. Under drought stress, 80% of WT seeds could germinate. In contrast, 86% seed germination was observed for *OsCyP2-P* transgenic plants. Kumari and coworkers observed also a significant difference in shoot and root growth of transgenic tobacco plants in comparison to WT plants. Under drought stress, shoot growth was decreased in transgenic and WT plants. However, the reduction in shoot length was lower in transgenic than in WT plants. Moreover, under drought conditions, high root growth was observed in transgenic tobacco plants, whereas wild-type roots were severely retarded [38]. In addition, *OsCyP21-4* upregulation of the cyclophilin localized in the Golgi apparatus was observed under drought stress and ABA treatment. Both factors caused a continuous upregulation of *OsCyP21-4* gene 24 h after treatment [87]. Gene expression and proteomic analysis showed that cyclophilin expression levels can differ between stress-tolerant and stress-susceptible cultivars of crop plants. Sharma and Singh showed that drought stress–induced expression of *SorgCyP20* is different between drought-tolerant and drought-susceptible cultivars of sorghum. The drought stress–induced *SorgCyP20* level was 3-fold higher in the leaves and 2.5-fold higher in seeds of drought-tolerant than in the drought-susceptible cultivars [88]. Differences in cyclophilins expression between drought-resistant and drought-sensitive wheat (*Triticum* aestivum) cultivars were also observed in comparative proteomic analysis. Chang and coworkers observed changes in two cyclophilin proteins level (GenBank Acc. Nos. gi|37788308, gi|37788308) in drought-tolerant wheat cultivar Xihan No. 2 in response to dehydration. Both of these peptidyl-prolyl *cis-trans* isomerases were classified into the group of proteins related to defense and protein translation, processing, and degradation. Both of these proteins showed similar expression during drought stress and rehydration. After 18 h of dehydration, expression increased and remained constant until 48 h when it increased again. An increase in expression was also observed after 24 h rehydration. Additionally, in the case of the drought-sensitive Longchun 23 variety, a change in the level of two proteins (GenBank Acc. Nos. gi|326499938, gi|242079005) belonging to the cyclophilin family was observed. The first one, assigned to the group of proteins responsible for metabolism and protein translation, processing, degradation, showed a similar expression pattern as described above. The second, assigned to the group of proteins involved in protein translation, processing, degradation and photosynthesis, was observed to be downregulated during all stages of drought stress and recovered after rehydration [89]. Since cyclophilins may participate in denatured proteins degradation as well as protein folding, they may be associated with stronger drought resistance in tolerant cultivar [63,64]. These findings also suggest that cyclophilins’ expression levels have an impact in stress tolerance on plants. In pigeon pea (*Cajanus cajan* L.), the cyclophilin gene (*CcCyP*) was shown to be stress inducible. The expression of this gene was upregulated in response to PEG and NaCl treatments. Interestingly, the overexpression of *CcCyP* in *A. thaliana* plants increased plant toleration to drought, evidenced by higher survival rates of a transgenic plant (~95%) as compared to WT plants (~60%) [66]. Furthermore, the upregulation of *ScCyP1, StCyP1,* *BrROC1-2* and *BrROC1-1* was observed under drought stress in *Solanum* *commersonii*, *Solanum tuberosum* and *Brassica rapa*, respectively [49,90,91].

### 3.2. Salinity Stress

Salinity is one of the strongest productivity-limiting factors in an environment, which affects plant vigor and germination [92]. High-salt concentration in soil affects plants in many ways, such as water stress (physiological drought), membrane disorganization, ion toxicity, oxidative stress [52,59]. The mechanism of salt tolerance in plants is not fully understood. Plants can adapt to salinity stress by expression profile change. Salinity stress was shown to have various impacts on the cyclophilins gene expression level. 

Increased expression of the *OsCyP21-4* gene encoding a cyclophilin localized in the Golgi apparatus of rice cells was observed under salinity stress. Furthermore, transgenic rice plants overexpressing *OsCyP21-4* were more tolerant to salt stress, compared to wild-type plants. Higher peroxidase activity was observed in the transgenic plants than in WT plants [87]. It was shown that the expression of *OsCyP20-2* increases under salinity stress [25,93]. Furthermore, ectopic expression *OsCyP20-2* in *A. thaliana* and *Nicotiana benthamiana* increase their tolerance to these stress factors. Moreover, salt tolerance increase was proportional to the increase in transgene expression [93]. Additionally, the *O. sativa* plant OsCyP2-P cyclophilin encoding gene is upregulated in response to salinity stress. Ectopic expression of *OsCyP2-P* increases the germination rate of overexpressing plants seeds (~96%), compared to WT plants (~76%). Under salinity stress, shoot and root growth were decreased in transgenic and WT plants. However, reduction in their length was lower in transgenic than in WT plants [38]. It was also shown that expression of the *A. thaliana AtCyP18-3* gene encoding cytosolic cyclophilin is elevated, whereas the *AtCyP19-4* gene coding ER localized protein is downregulated in response to salt stress [61]. The pigeon pea (*C. cajan* L.) cyclophilin gene (*CcCyP*) was shown to be upregulated in response to NaCl treatment. Furthermore, ectopic expression of *CcCyP* in *A. thaliana* plants increased plant tolerance to salinity stress [66]. Scholze and coworkers showed that the *Digitalis lanata* cyclophilin gene is upregulated under salt stress. [62]. Ectopic expression was also used to confirm the role of GhCyP1 cyclophilins in stress response. For instance, ectopic overexpression of *GhCyP1* gene in tobacco plant increases tolerance of *N. tabacum* to salinity stress [94]. A similar result was obtained after overexpression of the *Thellungiella halophile ThCyP1* gene in tobacco plants. Probably higher tolerance to salt stress was obtained, thanks to the regulation of appropriate folding of stress-related proteins by cyclophilins, or by the signal transduction processes mediated by these proteins [54]. Elevated expression of cyclophilin coding *StCyP1*, *BrROC1-2* and *BrROC1-1* were also observed in response to salinity stress in *S. tuberosum* and *B. rapa,* respectively [90,91].

### 3.3. Temperature Stress

Temperature stress in plants can be divided into three types: high, chilling, or freezing temperature. Temperature stress may cause low germination rates, inhibition of growth, limited photosynthesis and plant death. However, plants defend against temperature stress by regulating membrane lipid composition, stress-related transcription factors, and metabolite synthesis [51]. Some reports revealed that cyclophilins also participate in plant response to temperature stress. The expression of *AtCyP19-2* and *AtCyP20-2* genes encoding cyclophilin localized, respectively, in the cytoplasm and chloroplast was shown to be upregulated in response to cold treatment [61,95]. On the contrary, the expression level of *AtCyP18-1* gene was elevated in response to high temperature [55]. In *O. sativa* plants, the OsCyP2-P cyclophilin encoding gene is upregulated in response to high temperature. Ectopic expression of *OsCyP2-P* also increases of tolerance transgenic tobacco plants to high temperature [38]. *C. cajan* L. cyclophilin gene (*CcCyP*) was shown to be upregulated in response to elevated (37 and 42 °C) and lowered (4°C) temperatures. Furthermore, these findings were supported by ectopic expression of *CcCyP* in *A. thaliana* plants, which increases plant toleration to extreme temperatures [66]. The upregulation of cyclophilin genes was also observed in the *Solanaceae* family. For instance, *S. commersonii* and *S. tuberosum* cyclophilin gene expression level increased in response to low temperature [49,90]. In *B. rapa*, two cyclophilin encoding genes were identified as being stress inducible (*BrROC1-2, BrROC1-1*). Their expression increased in response to high or low temperature [91].

### 3.4. Light Stress

Light is one of the most important environmental factors affecting plant growth and development as well as photosynthesis [96]. However, light deficiency and excess can be harmful to plants. Cyclophilins are a group of proteins whose expression changes in response to light stress. Chloroplast cyclophilin AtCyP20-2 was shown to participate in high light stress response in *A. thaliana* [97]. It was shown that AtCyP20-2 takes part in the process of formation and maintenance of the NAD(P)H dehydrogenase (NDH) subcomplex [56], which prevents over-reduction of stroma [98]. Additionally, in *O. sativa,* expression of the *OsCyP20-2* gene increased under light stress [93]. Furthermore, ectopic expression *OsCyP20-2* in *A. thaliana* and *Nicotiana benthamiana* also increased their tolerance to these stress factors. Kim and coworkers suggested that increases in the light tolerance in *A. thaliana* is related to higher PPIase activity in transgenic plants. Interestingly, the highest *OsCyP20-2* expression was consistent with the highest tolerance [93]. The role of *A. thaliana* cyclophilins in light stress response was also demonstrated by the analysis of knock-out mutant plants. For example, loss-of-function mutants of *atcyp20-3* appeared to be more sensitive to light stress [99]. Cyclophilin CyP20-3 was reported to promote plastids SAT1 (serine acetyltransferase 1), assisting the folding or assembly of SAT1enzyme to form the well-known hetero-oligomeric complex [65] and thus, cysteine and glutathione production under high light conditions [99].

### 3.5. Oxidative Stress

Oxidative stress is a complex chemical and physiological change that accompanies biotic and abiotic stresses in plants. Oxidative stress develops as a result of the production and accumulation of reactive oxygen species (ROS). Cyclophilin was also shown to be a possible element of the oxidative stress response. Transgenic rice plants overexpressing *OsCyP21-4* were more tolerant to hydrogen peroxide treatment, as compared to WT plants. Higher peroxidase activity was observed in the transgenic plants than in WT plants [87]. The role of *A. thaliana* cyclophilins in oxidative stress response was demonstrated by the analysis of knock-out mutant of *atcyp20-3,* whose tolerance to oxidative stress was lower in comparison to WT plants [99]. Furthermore, CyP20-3 regulates cellular redox homeostasis. For example, OPDA (cis-(+)-12-oxo-phytodienoic acid) promotes CyP20-3 to form a complex with serine acetyltransferase 1. These complexes activate the formation of a hetero-oligomeric cysteine synthase complex with *O*-acetylserine(thiol)lyase B in chloroplasts. The cysteine synthase complex then activates sulfur assimilation, which leads to increased levels of thiol metabolites and creates a cellular reduction potential [67]. Strong support for the thesis that cyclophilins may take part in the response to oxidative stress is research that used the ectopic expression of *OsCyP20-2* in *A. thaliana* and *Nicotiana benthamiana,* which resulted in an increase in transgenic plants’ tolerance to oxidative stress induced by methyl viologen [93].

### 3.6. Other Stress Factors

Change in the cyclophilin genes’ expression level was also presented in several other reports. For example, increasing expression of *CaCyP1* in *Capsicum annuum* was observed after abiotic stress elicitor treatment, such as salicylic and jasmonic acids [100]. Furthermore, Scholze and coworkers showed that the *Digitalis lanata* cyclophilin gene is upregulated by PbNO_3_ treatment [62]. It was also shown that the cyclophilin gene expression level increases in response to wounding in *S. commersonii* [49]. Nitrogen starvation was also shown as a stress factor that can cause cyclophilins coding genes upregulation in *N. tabacum* [101].

## 4. Cyclophilin Function in Plant–Microbe Interactions 

### 4.1. Pathogenic Plant–Microbe Interactions

Plants have evolved a set of host defense mechanisms to combat infection against a wide variety of pathogens. This is often connected with so-called plant resistance proteins (R-proteins) that specifically recognize pathogen effectors delivered into plant cells during infection. These proteins generate resistance, resulting in a common set of host defense responses, such as transcriptional reprogramming and localized cell death at the site of infection [102,103]. In order to enable plant colonization, pathogenic bacteria, such as *Pseudomonas syringae,* deliver to the plant cell via the type III secretion system (TTSS), virulence factors (effector proteins) that subvert host defenses. Effectors modulate the plant physiology in order to sustain pathogen growth [104]. One of the effector proteins delivered by *P. syringae* to the infected cell is AvrRpt2, which in bacteria exists as an inactive cysteine protease [105]. Effectors may need to remain unfolded in bacteria in order to transverse the TTSS. After delivery, AvrRpt2 is activated and able to self-process and cleave its N-terminal 71 amino acids, which encode a signal for TTSS delivery into plant cells. It was shown in *A. thaliana* cells that single-domain cyclophilin (ROC1) functions as an activator of the AvrRpt2, which is rich in Gly-Pro motifs [106]. Coaker and coworkers indicated in their work that AvrRpt2 is activated by the host cyclophilin, which directly binds to AvrRpt2 at four sites and properly folds the effector by prolyl isomerization [107]. They have chosen four possible Gly-Pro motives (Gly137Pro138Arg139Leu140; Gly151Pro152Ala153Gly154; Gly194Pro195Iso196Iso197; Gly230Pro231Asp232Leu233) and performed site-directed mutagenesis of each Gly residue preceding the proline to Ala. mutation in all Gly residues (Gly137Gly151Gly194Gly230) abolished the interaction with ROC1 in vitro. All four cyclophilin binding sites are located within or in close proximity to the catalytic center of AvrRpt2. These results indicate that the isomerization of multiple peptide bonds within AvrRpt2 between *trans* and *cis* conformation controls the activation of this bacterial effector protein. After activation, the AvrRpt2 protease cleaves RIN4 protein and the elimination of RIN4 activates RPS2 (R-protein) and defense responses [108]. Recent experiments presented by Pogorelko and coworkers have shown that not only ROC1 cyclophilin from *A. thaliana* is involved in pathogen resistance [109]. They identified two cyclophilins, AtCyP19 localized in the cytoplasm and AtCyP57 localized in the cytoplasm and nucleus, as potential proteins involved in response to *P. syringae* infection. After bioinformatics analysis, the authors indicate that the expression of these two *A. thaliana* cyclophilin genes increased more than 2-fold during biotic stress. To confirm this bioinformatic analysis, they used quantitative RT-PCR to check the expression levels of *AtCyP19*, and *AtCyP57* in response to *P. syringae* infection. The authors discovered that knock-out mutations of these two *A. thaliana* cyclophilins result in increased susceptibility to *P. syringae*, whereas overexpression of these genes alters the transcription profile of pathogen-related defense genes and leads to enhanced resistance. AtCyP19 was shown to be involved in reactive oxygen species production, and AtCyP57 provided callose accumulation in the plant cell wall. Constitutive expression of *AtCyP19* resulted in increased accumulation of reactive oxygen species (ROS), which inhibit pathogen growth. Indeed, ROS were proposed to act as primary signaling molecules in abiotic and biotic stresses, activating mitogen-activated protein kinase (MAPK) pathways [110]. The authors discovered that *AtCyP19* expressing plants showed increased expression of *MEKK1*, which is known to play an important role in MAPK pathways during the pathogen attack [111]. The sub-cellular localization of AtCyP57 indicates that this protein is present in the cell nucleus and could participate directly in the regulation of certain genes, such as *PAD4* (coding for Phytoalexin Deficient Protein 4), which is activated downstream of MEKK1, causing downstream WRKY33 (WRKY DNA-binding protein 33) induction. WRKY33 is a member of a large WRKY transcription factors family in *A. thaliana* [112] and represents self-controlling pathogen-inducible transcription factors [113,114], which are also involved in the SA-dependent signaling pathway [115]. As a consequence, *bGS2* (coding for the β-glucan synthase 2) expression is induced, which is required for callose production.

Overexpression of cotton cyclophilin GhCyP1 performed in transgenic tobacco conferred higher tolerance to salt stress but also to *Psuedomonas syringae* pv. *tabaci* infection as compared with control plants [94]. Information about plant–microbe responsive cyclophilins are collected in Table 2.

Different classes of antifungal proteins isolated from various plants include chitinases, cyclophilins (CyPs), defensins, lectins and lipid transfer proteins. CyP-like antifungal proteins were isolated from black-eyed pea, mung bean, Chinese cabbage and chickpea [116,117,118]. The recombinant pgCyP from *Panax ginseng* possesses PPIase activity and exhibits strong antifungal activity against *Phytophthora cactorum* [119].

Molecular studies on host–pathogen interactions have revealed that some pathogenic bacteria exploit host cell proteins for their benefit. *Agrobacterium tumefaciens* is a plant pathogen that is able to transfer DNA fragment (T-DNA) from its tumor-inducing (Ti) plasmid into a plant cell and integrate it with host genomic DNA. As a consequence of this, the expression of oncogenes coded in T-DNA leads to the overproduction of plant growth hormones, resulting in tumor formation [120]. The T-complex consists of a single-stranded T-DNA with covalently bound to its 5′ endonuclease VirD2, and single-stranded DNA binding proteins VirE2. Both proteins have nuclear localization signals and play important functions during the T-DNA transfer and its integration with the host genome. Deng and coworkers, using the yeast two-hybrid system, identified two isoforms of *A. thaliana* cyclophilins, which interact with VirD2 endonuclease [121]. They also discovered that the VirD2–cyclophilin complex can be disrupted in vitro by CsA, which inhibits *Agrobacterium*-mediated transformation of *A. thaliana*. Authors suggest that host cyclophilins play an important function in T-DNA transfer. Cyclophilins may play a role in maintaining the correct conformation of VirD2; however, van Kregten and coworkers in 2009 discovered that the VirD2 cyclophilin-binding domain is not necessary for transformation and suggest that cyclophilins may not be required for VirD2 function in the plant cell [122]. 

### 4.2. Beneficial Plant–Microbe Interactions

Another type of plant microbe–host interaction is symbiosis. Symbiotic and pathogenic microorganisms have in common that they live in or on host organisms or host cells. Despite the different forms of interactions, symbiotic and pathogenic microorganisms have in common that they are adapted to a particular environmental niche represented by the host organism.

*Lupinus luteus* cytosolic cyclophilin (CyPA) is highly expressed in root nodules [24]. The formation of the root nodule is a consequence of the symbiotic interaction between legumes with symbiotic soil bacteria collectively named rhizobia. Nodulation involves an enhanced expression of certain plant genes, which are referred to as early- or late-nodulin genes [123]. Many nodulin genes encode hydroxyproline-rich glycoproteins (HRGP) and proline-rich proteins (PRP) [124,125,126,127]. Some of them are cell wall components affecting nodule formation and function. The amount of *CyP* transcripts is dramatically increased in root nodules. In situ hybridization experiments indicate that *CyP* transcripts are localized mainly in meristematic tissues with the highest level observed in the nodule meristem zone. The authors have shown that infection of the plant by the symbiotic bacteria *Bradyrhizobium* sp. (lupinus) and the subsequent nodule development enhances the expression of *CyP* genes, especially in the root meristem zone. The promoter of *LlCyP* was also analyzed in the model plant *Lotus japonicus,* using site-directed mutagenesis [27]. *L. japonicus* and *Lupinus luteus* produce different types of nodules, determinate and indeterminate, respectively. In contrast to indeterminate nodules, the determinate nodules do not have a strictly defined meristematic zone [128,129]. The activity of *LlCyP* promoter in nodules is probably connected with the need for a large amount of cyclophilins that carry out isomerization around Xaa-Pro bonds, in the protein folding enabling HRGPs and PRPs to attain their native structure [24]. Aloui and coworkers reported that, using proteomic analysis of *Medicago truncatula* roots infected with symbiotic fungus *Glomus intraradices,* among 26 mycorrhiza-related proteins that were identified, there was a cyclophilin [28]. Additionally, they showed that its expression was also upregulated in the presence of heavy metals, such as Cd. 

## 5. Conclusions

Cyclophilins are multi-functional proteins that regulate many processes in plant growth and development, hormone signaling, abiotic stress response and plant–microbe interactions. Cyclophilins can be found in all subcellular compartments. Various plant cyclophilins are encoded by numerous genes, revealing functional differentiation. Most of the information about cyclophilin structure and function comes from the bioinformatic analysis of the existing data or from the NGS (transcriptome) and proteome analyses mainly for model plants. As stress conditions negatively affect the growth and development of plants, which drastically affects the yield of crops, a good direction for the use of these data is the preparation of transgenic plants resistant to a given stress factor. The plant–microbe interaction is not only pathogenesis, but also beneficial. In contrast to pathogenesis, symbiotic interaction has many benefits, and it is worth analyzing the proteins involved in this process. We still have insufficient information on the specific function of cyclophilins, so more research is needed to answer this question.

## Figures and Tables

**Table 1:** Abiotic stress-responsive cyclophilins. The domain architecture diagrams were prepared using IBS software [50].

Protein Name	Organism	Stress Factor	Expression Response to Stress Factor	Subcellular Localization	Domain Architecture	References
**AtCyP18-1**	*Arabidopsis thaliana*	heat	up-regulation	cytoplasm	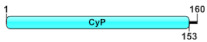	[51]
**AtCyP18-3**	*Arabidopsis thaliana*	salinity	up-regulation	cytoplasm	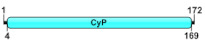	[52]
**AtCyP19-1**	*Arabidopsis thaliana*	drought	up-regulation	cytoplasm, nucleus	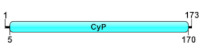	[53]
**AtCyP19-2**	*Arabidopsis thaliana*	temperature	up-regulation	cytoplasm	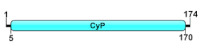	[54]
**AtCyP19-4**	*Arabidopsis thaliana*	salinity	down-regulation	ER	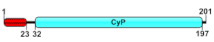	[52]
**AtCyP20-2**	*Arabidopsis thaliana*	light temperature	up-regulation	chloroplast tylakoid lumen	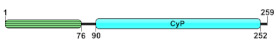	[54,55]
**AtCyP** **20-3**	*Arabidopsis thaliana*	light oxidative	Decreased stress tolerance in knock-out mutants.	chloroplast	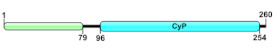	[56]
**AtCyP** **21-2**	*Arabidopsis thaliana*	drought	Decreased stress tolerance in knock-out mutants.	ER	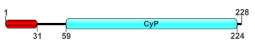	[57]
**OsCyP2-P**	*Oryza sativa*	drought, salinity, temperature	upregulated; Ectopic expression increases stress tolerance in mutants.	cytoplasm and nucleus	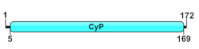	[36]
**OsCyP18-2**	*Oryza sativa*	drought	upregulated; Overexpression increases stress tolerance.	nucleus	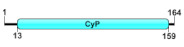	[58]
**OsCyP20-2**	*Oryza sativa*	salinity light oxidation	upregulated; Ectopic expression increases stress tolerance in mutants.	chloroplast/ tylakoid lumen	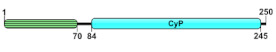	[25,59]
**OsCyP21-4**	*Oryza sativa*	drought salinity oxidation	upregulated	Golgi apparatus	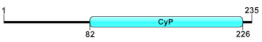	[60]
**GhCyP1**	*Gossypium Herbaceum*	salinity	Ectopic expression increases stress tolerance.	cytoplasm	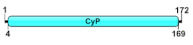	[61]
**ThCyP1**	*Thellungiella halophile*	salinity	Ectopic expression increases stress tolerance.	nucleus	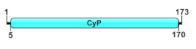	[62]
**CcCyP**	*Cajanus cajan*	temperature salinity osmotic drought	upregulated; Ectopic expression increases stress tolerance in mutants.	nucleus	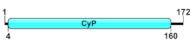	[63]
**ScCyP1**	*Solanum commersonii*	drought	upregulated	-	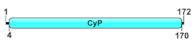	[47]
**StCyP1**	*Solanum tuberosum*	drought salinity	upregulated	cytoplasm	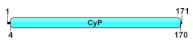	[64]
**CaCyP1**	*Capsicum annuum*	jasmonic acid, salicylic acid (abiotic stress elicitors)	upregulated	-	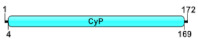	[65]
**BrROC1-1** **BrROC1-2**	*Brassica rapa*	drought salinity temperature	upregulated	-	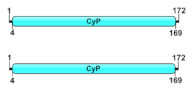	[66]
**NtCyP2**	*Nicotiana tabacum*	nitrogen starvation	upregulated	cytoplasm	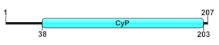	[67]
**SorgCyP20**	*Sorghum bicolor*	drought	upregulated	cytoplasm	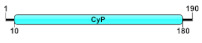	[68]

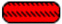
 ER targeting signal; 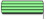 thylakoid lumen targeting signal; 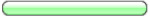 chloroplast targeting signal; 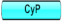 cyclophilin domain; “-” not detected.

**Table 2:** Plant–microbe responsive cyclophilins. The domain architecture diagrams were prepared using IBS software [50].

Protein Name	Organism	Stress Factor	Expression Response to Stress Factor	Subcellular Localization	Domain Architecture	References
**AtCyP18-3 (ROC1)**	*Arabidopsis. thaliana*	*Psuedomonas syringae* infection	upregulated	cytoplasm	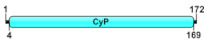	[103]
**AtCyP19**	*Arabidopsis. thaliana*	*Psuedomonas syringae* infection	upregulated	cytoplasm	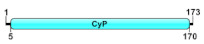	[106,107,108]
**AtCyP57**	*Arabidopsis. thaliana*	*Psuedomonas syringae* infection	upregulated	cytoplasm and nucleus	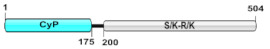	[106,107,108]
**GhCyP1**	*Gossypium Herbaceum*	*Psuedomonas syringae* pv. *Tabaci* infection	upregulated	cytoplasm	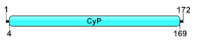	[61]

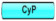
 Cyclophilin domain 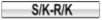 Arg/Lys rich domain.

## Data Availability

Not applicable.
